# Ultra-lightweight robotic hip exoskeleton with anti-phase torque symmetry for enhanced walking efficiency

**DOI:** 10.1038/s41598-025-95599-2

**Published:** 2025-03-29

**Authors:** Bokman Lim, Byungjune Choi, Changhyun Roh, Jewoo Lee, Yong-Jae Kim, Younbaek Lee

**Affiliations:** 1 Robot R&D Team, WIRobotics, Yongin, 16942 Korea; 2 School of Electrical, Electronics & Communication Engineering, Korea University of Technology and Education, Cheonan, 31253 Korea

**Keywords:** Rehabilitation, Mechanical engineering

## Abstract

This paper presents a novel robotic exoskeleton that is exceptionally lightweight and compact, while providing effective gait assistance. To maximize the system’s assistance-to-weight/size ratio, the design focuses on two key aspects of human gait mechanics: (1) the contribution of the hip joints to power generation, and (2) the symmetrical nature of hip flexion and extension torques during walking. Based on these principles, we developed a compact hip exoskeleton with a single actuator. This actuator simultaneously drives the hip joint in the sagittal plane, facilitating both flexion and extension during gait. An adaptive delayed output feedback controller was implemented, ensuring consistent performance across diverse walking conditions using a single rotational sensor and actuator. To evaluate the exoskeleton’s effectiveness, a 4-week outdoor walking exercise program was conducted with nine elderly participants. Their gait, balance, and muscle strength were measured before and after the program to assess improvements. Results showed significant improvements in walking speed (14.8% in the 10-m walk and 10.6% in the 6-min walk), as well as enhanced performance in the timed up-and-go test (24.5%) and the short physical performance battery test (18.7%). Ankle dorsiflexion and plantar flexion muscle strength increased by 75.45% and 45.8%, respectively. Additionally, metabolic measurements from three young adults indicated a 13.6 ± 3.2% reduction in the net metabolic cost of walking with the exoskeleton compared to walking without it. These results demonstrate that the single actuator-based hip exoskeleton offers effective gait assistance while maintaining a lightweight and compact design, highlighting its potential for widespread use in various applications.

## Introduction

Recent advancements in robotic exoskeletons have significantly reduced walking energy consumption while providing effective assistance across diverse populations^1–4^. These developments have enhanced walking abilities in groups such as the elderly^5^ and individuals with conditions like stroke^6^ or Parkinson’s disease^7^. However, exoskeletons are still primarily used for rehabilitation in controlled environments, such as laboratories and hospitals. To be practical for everyday use, such as supporting seniors’ exercise, aiding workers’ mobility, or serving as travel devices, exoskeletons must undergo substantial advancements in compactness, weight, and usability, akin to the evolution of mobile phones, which have become lighter, more compact, and versatile.

**Fig. 1:**
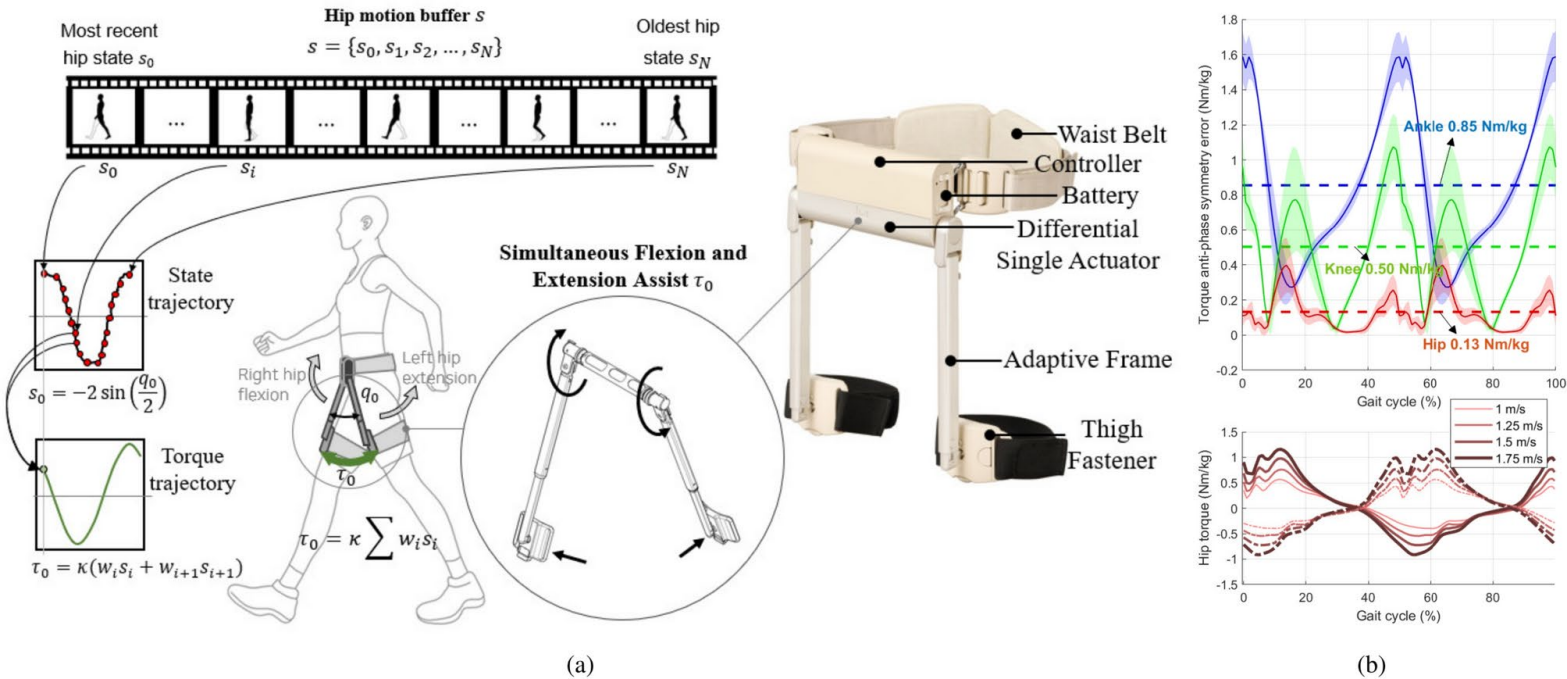
Gait assistance strategy using hip torque anti-phase flexion-extension symmetricity. (**a**) Single actuator-based hip exoskeleton WIM and its hip assistance strategy. (**b**) Comparison of torque symmetricity error between the hip, knee, and ankle joints during walking.

Focusing on hip joint assistance offers distinct advantages by contributing significantly to positive power generation during walking while also addressing key engineering challenges. The hip and ankle muscles are both crucial in generating positive power during walking. At a walking speed of 1.25 m/s on level ground, the hip, knee, and ankle contributes 37%, 19%, and 44%, respectively^8^. When walking uphill at a 5.71-degree incline, these contributions shift to 47%, 19%, and 34%, respectively^8^. During gait initiation (the first two walking steps), the hip contribution reaches 60%, with the knee at 28% and the ankle at 12%^9^. This high hip contribution during uphill walking and gait initiation underscores the importance of hip assistance, particularly for individuals with walking impairments, such as those with Parkinson’s disease, who experience difficulty in initiating gait^7^. In aging populations, joint power redistribution results in higher hip power contributions. For example, elderly individuals exhibit 48% hip, 19% knee, and 33% ankle contributions during level walking^10^. This shift highlights the utility of hip exoskeletons in reducing metabolic energy consumption and improving gait performance following exoskeleton-assisted exercise^5^. Although the ankle is a major contributor to positive power, it is highly sensitive to added weight. A 4-kg load on the foot elevates the metabolic rate by 36% compared to no load^11^, and the metabolic impact is amplified at higher walking speeds. During running, ankle weight increases the metabolic rate nearly three times more than hip weight^12^. From an engineering perspective, the ankle’s sensitivity to weight necessitates greater emphasis on weight reduction in distal devices. Actuators are often placed near the waist to minimize this impact, but this leads to power transmission losses and challenges in maintaining compact system designs.

Drawing on two key principles of human gait mechanics: (1) the increasing role of hip joints in power generation with age^10^, and (2) the symmetricity of flexion and extension torques in the hip joints during walking, we propose a lightweight, single-actuator hip exoskeleton designed for gait assistance. This compact design, combined with a novel control strategy reliant on minimal sensors and actuators, is expected to offer unprecedented user convenience and lightness (Supplementary Fig. S1). During normal walking, the hip and ankle joints are the primary contributors to positive power generation, with hip power playing a significant role in conditions requiring walking assistance, such as elderly walking^10^, uphill walking^8^, and gait initiation^9^. Our focus also includes the symmetry of left and right hip torque trajectories. Hip anti-phase flexion-extension symmetry refers to a characteristic of human gait in which the hip joints of each leg move in opposite directions in a coordinated manner. During walking, as the hip joint of one leg flexes or bends, the hip joint of the opposite leg extends or straightens. This alternating pattern of flexion and extension stabilizes the body and maintains balance during walking. Figure 1b illustrates the hip joint torque trajectories at various walking speeds, revealing the anti-phase flexion-extension symmetry of hip torques. This finding suggests that a single actuator can effectively assist both hip flexion and extension simultaneously during walking (Fig. 1a), a feature not observed in the knee and ankle joints (Supplementary Fig. S2). Most prior research has analyzed hip, knee, and ankle trajectories without specifically addressing the coordination between the left and right sides, except for a recent study focused on gait initiation dynamics^9^.

Existing hip exoskeletons for walking assistance commonly utilize a dual-actuators design, with separate actuators positioned at the left and right hip joints to assist each joint individually^4,13–15^. Previous single-actuator-based exoskeletons for gait assistance have primarily provided unilateral support, such as assisting only in hip flexion^16^ or hip extension^17^. In contrast, dual-motor devices positioned at the hip can deliver bidirectional flexion-extension assistance^4,5^. To maintain a compact design with a single actuator, innovative approaches are required in drive transmission, sensing, and actuation. Using multiple sensors and high computational power to control assistive torque would inevitably increase the system’s complexity, size, and weight, undermining the goal of creating a lightweight, user-friendly device. In practice, finding an exoskeleton with an active actuator weighing less than 2 kg remains a significant challenge^2,4,15,18–23^ (Supplementary Fig. S1).

**Fig. 2:**
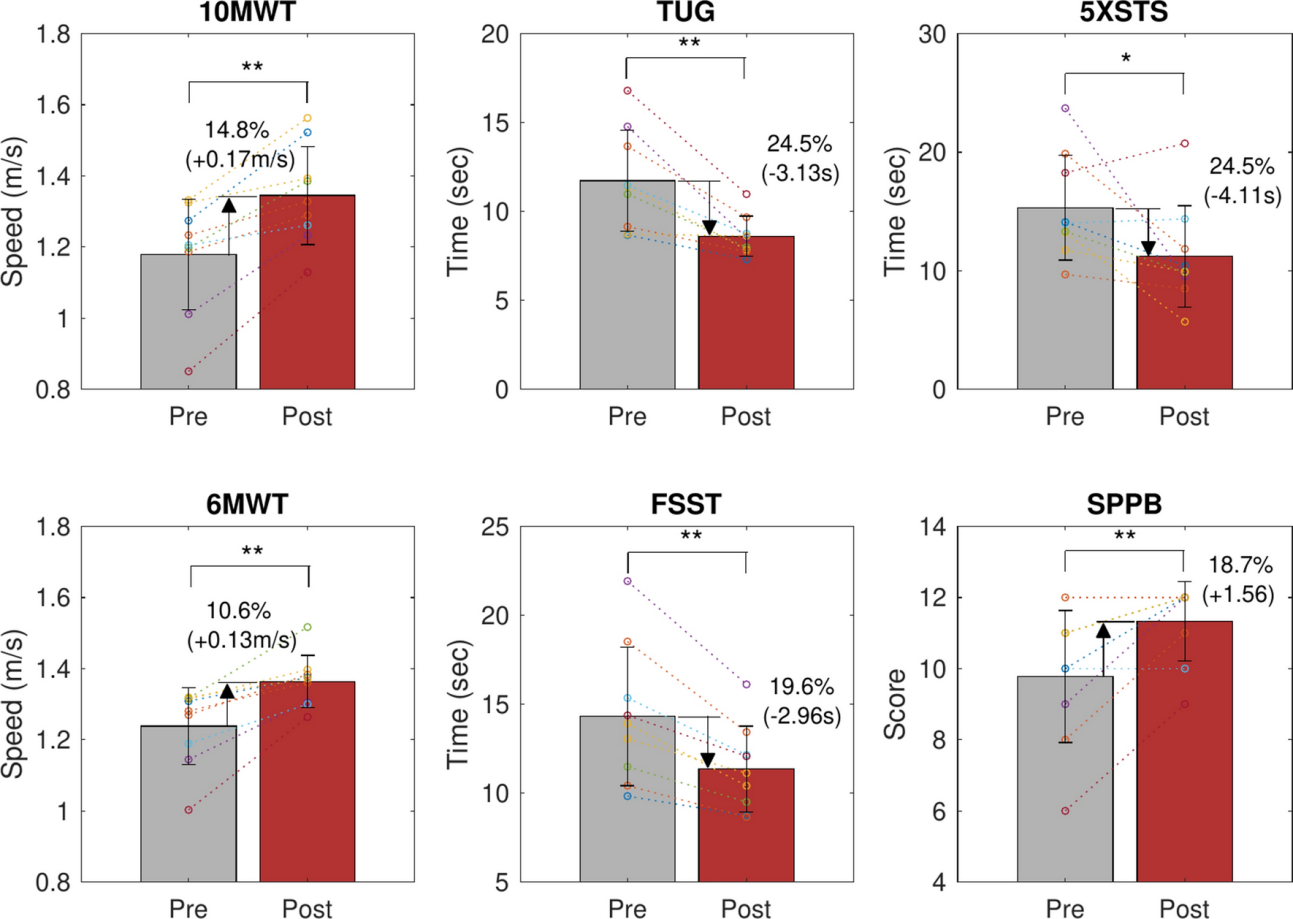
Changes in gait quality measurements before gait exercise (Pre) and after gait exercise sessions (Post). *p-value < 0.05, **p-value < 0.01 for paired t-test comparisons between Pre and Post conditions.

**Table 1:** Changes in gait quality measurements before gait exercise (Pre) and after gait exercise sessions (Post). Mean ± SD; *p-value < 0.05, **p-value < 0.01 for paired t-test comparisons between Pre and Post conditions.

	Pre	Post	Post-Pre	*p*-value
10MWT (m/s)	1.18 ± 0.16	1.34 ± 0.14	0.17 ± 0.09**	0.0004
6MWT (m/s)	1.24 ± 0.11	1.36 ± 0.07	0.13 ± 0.07**	0.001
TUG (s)	11.73 ± 2.84	8.60 ± 1.13	– 3.13 ± 2.04**	0.002
FSST (s)	14.32 ± 3.90	11.35 ± 2.41	– 2.96 ± 1.60**	0.001
5xSTS (s)	15.32 ± 4.41	11.20 ± 4.27	– 4.11 ± 5.08*	0.041
FRT (cm)	22.8 ± 7.47	28.0 ± 5.50	5.17 ± 6.75	0.051
SPPB (score)	9.78 ± 1.86	11.33 ± 1.12	1.56 ± 1.24**	0.005

To enhance the versatility of this single-actuator design, we propose an adaptive delayed output feedback control (Adaptive DOFC) algorithm. This algorithm adapts to various gait patterns using only a single sensor and actuator, generating smooth and immediate interaction torques for both assistance and resistance. Unlike recognition-based control methods, which depend on gait recognition performance^24–29^ the Adaptive DOFC algorithm adjusts control parameters based on real-time state-trajectory fluctuations, enabling immediate responses to different walking speeds (refer to the Supplementary Method). The device assists both left and right hip flexion and extension using a single actuator and can adapt to asymmetrical walking conditions by incorporating an asymmetry factor, further enhancing its versatility (Supplementary Figs. S3, S4).

This study aims to demonstrate the effectiveness of the proposed hip exoskeleton in delivering gait assistance while maintaining a lightweight and compact design. To this end, we conducted a four-week exercise program with elderly participants and performed pre- and post-functional assessments. We sought to determine whether the lightweight, single-motor hip exoskeleton could achieve improvements in gait performance comparable to devices using dual actuators for bilateral hip flexion-extension assistance^5^. Additionally, metabolic energy consumption experiments were conducted with young adults to assess the extent to which the exoskeleton reduced energy expenditure during walking. To our knowledge, this is the first ultra-lightweight hip exoskeleton, weighing 1.6 kg, that enhances the gait performance of elderly individuals through an outdoor walking exercise program and reduces metabolic energy consumption by more than 10% compared to walking without the device (Supplementary Fig. S1). Further, we discuss various additional usability advantages of this system.

## Results

### Anti-phase hip torque symmetry

We compared the anti-phase symmetry error by analyzing the hip flexion/extension, knee flexion/extension, and ankle dorsiflexion/plantarflexion torque trajectories during a walking gait cycle at various speeds. As shown in Fig. 1b, the anti-phase torque symmetry errors for the hip, knee, and ankle joints are 0.13 Nm/kg, 0.5 Nm/kg, and 0.85 Nm/kg, respectively, with the hip torque showing a relatively smaller error value. Despite variations in walking speeds, specifically 1 m/s, 1.25 m/s, 1.5 m/s, and 1.75 m/s, hip torque anti-phase symmetry is consistently maintained during gait. This finding indicates that the torque symmetry characteristic can be effectively utilized in hip exoskeleton devices designed to assist with hip joint torque.

**Fig. 3:**
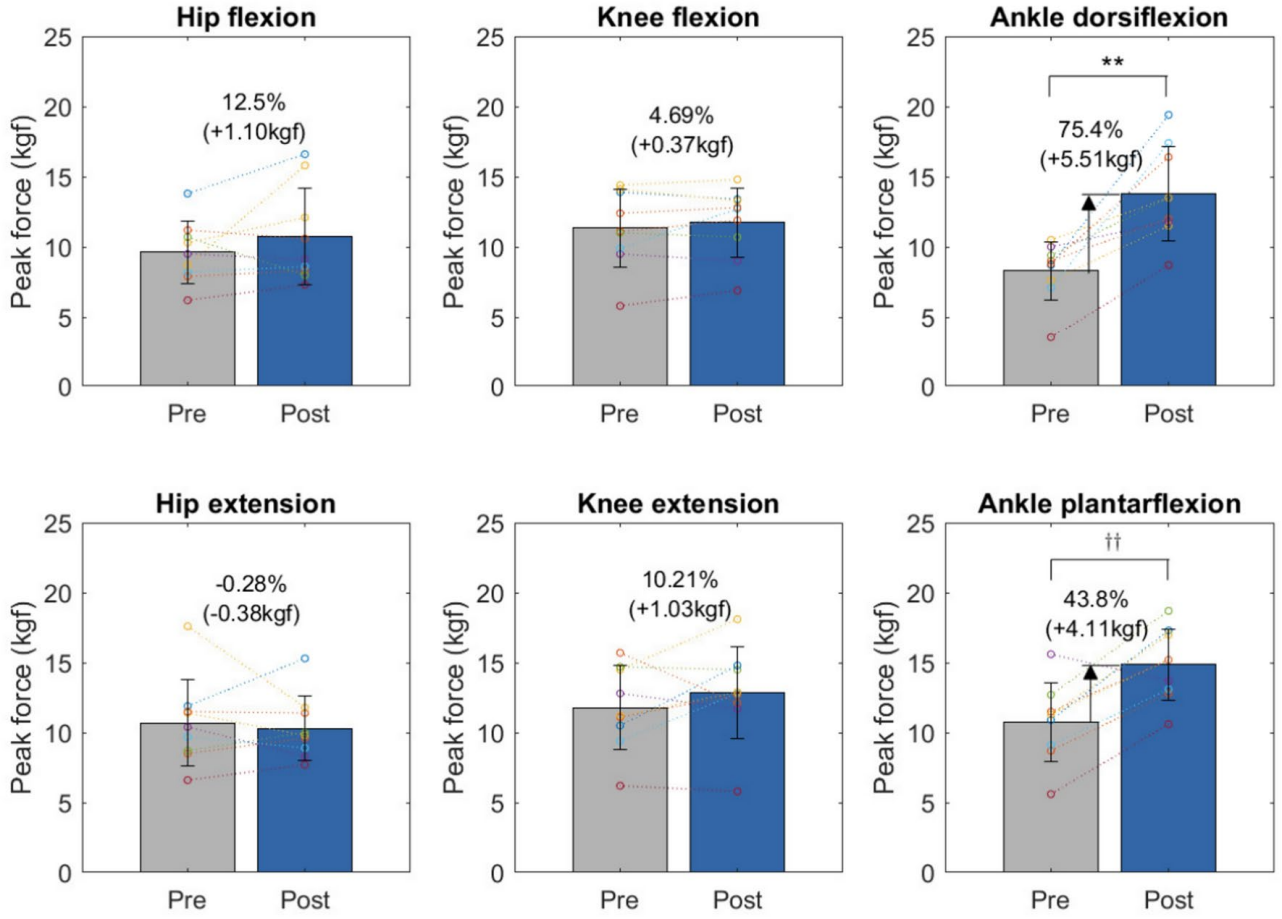
Changes in muscle strengths before gait exercise (Pre) and after gait exercise sessions (Post). **p-value < 0.01 for paired t-test (Pre vs. Post), $^{\dagger \dagger }$ *p*-value < 0.01 for Wilcoxon signed-rank test (Pre vs. Post).

**Table 2:** Changes in muscle strength measurements before gait exercise (Pre) and after gait exercise sessions (Post).

	Pre	Post	Post-Pre	*p*-value
Hip flexion (kgf)	9.58 ± 2.21	10.68 ± 3.44	1.10 ± 2.74	0.263
Hip extension (kgf)	10.66 ± 3.10	10.28 ± 2.29	– 0.38 ± 2.63	0.673
Knee flexion (kgf)	11.33 ± 2.75	11.69 ± 2.46	0.37 ± 1.13	0.357
Knee extension (kgf)	11.77 ± 2.98	12.81 ± 3.27	1.03 ± 2.57	0.261
Ankle dorsiflexion (kgf)	8.27 ± 2.08	13.78 ± 3.35	5.51 ± 3.27**	0.001
Ankle plantarflexion (kgf)	10.71 ± 2.79	14.81 ± 2.57	4.11 ± 2.47$^{\dagger \dagger }$	0.008

Mean ± SD; **p-value < 0.01 for paired t-test (Pre vs. Post), $^{\dagger \dagger }$ *p*-value < 0.01 for Wilcoxon signed-rank test (Pre vs. Post).

### Effects on gait quality

Figure 2 and Table 1 present the gait quality measurement results of the participants before (Pre) and after (Post) performing the designated exercise program. Most gait quality indicators exhibited significant improvements upon completion of the exercise program. The 10-meter walking test (10MWT) walking speed increased from an average of 1.18 ± 0.16 m/s to 1.34 ± 0.14 m/s, reflecting a 14.8% improvement, with an increase of 0.17 ± 0.19 m/s in walking speed (mean ± SD). Similarly, the 6-minute walking test (6MWT) walking speed increased from 1.24 ± 0.11 m/s to 1.36 ± 0.07 m/s, indicating a 10.6% improvement with an increase of 0.13 ± 0.07 m/s. The Timed Up and Go (TUG) test time was reduced from 11.73 ± 2.84 s to 8.60 ± 1.13 s, indicating a 24.5% improvement with an average reduction of 3.13 ± 2.04 s. The Four Square Step Test (FSST) completion time decreased from 14.32 ± 3.90 s to 11.35 ± 2.41 s, a 19.6% improvement with an average reduction of 2.96 ± 1.60 s. The Five Times Sit-to-Stand Test (5xSTS) completion time was reduced from 15.3 ± 4.41 s to 11.20 ± 4.27 s, showing a 24.5% improvement with an average reduction of 4.11 ± 5.08 s. Although the Functional Reach Test (FRT) distance increased from 22.8 ± 7.47 cm to 28.0 ± 5.50 cm, this change was not statistically significant. However, the Short Physical Performance Battery (SPPB) score increased from 9.78 ± 1.86 to 11.33 ± 1.12, reflecting an 18.7% improvement with an increase of 1.56 ± 1.24 points.

**Fig. 4:**
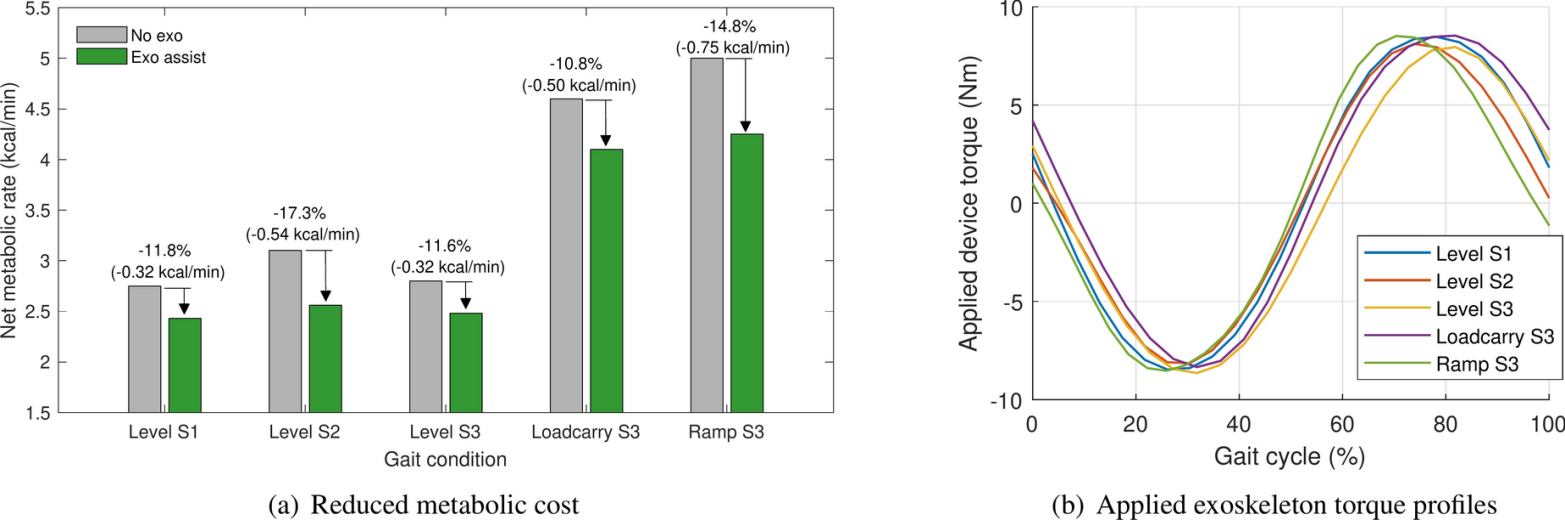
Reduced metabolic cost and applied torque during level, load-carrying, and uphill treadmill walking.

### Effects on lower limb muscle strength

Figure 3 and Table 2 show the muscle strength measurements (manual muscle testing [MMT]) of the participants by lower limb joint before (Pre) and after (Post) the designated exercise program. After completing the exercise program, significant improvements were observed in the ankle joint strength. Ankle dorsiflexion strength increased from 8.27 ± 2.08 kgf to 13.78 ± 3.35 kgf, reflecting a 75.4% improvement with an increase of 5.51 ± 3.27 kgf. Ankle plantarflexion strength increased from 10.71 ± 2.79 kgf to 14.81 ± 2.57 kgf, representing a 43.8% improvement with an increase of 4.11 ± 2.47 kgf. Hip flexion, knee flexion, and knee extension strengths increased by an average of 1.10 ± 2.74 kgf, 0.37 ± 1.13 kgf, and 2.57 kgf, respectively, while hip extension strengths decreased from 10.66 ± 3.10 kgf to 10.28 ± 2.29 kgf, resulting in a decrease of 0.38 ± 2.63 kgf. Proximal hip and knee strength showed minimal change after participating in the gait-assisted exercise program, while distal ankle muscle force significantly increased.

### Effects on gait efficiency (supplemental study, n = 3)

The device, including the battery and the waist and thigh fasteners, weighed a total of 1.6 kg. The hip exoskeleton reduced the metabolic cost of walking for the three participants by 10.8–17.3%, as shown in Fig. 4a. The applied exoskeleton torque profiles of the device are shown in Fig. 4b. The net metabolic cost of level treadmill walking with the hip exoskeleton (2.48 ± 0.05 kcal/min) was 13.6 ± 3.2% lower compared to walking without the exoskeleton (2.88 ± 0.19 kcal/min) (Supplementary Table S1).

## Discussion

Our main finding is that the proposed single-actuator exoskeleton design effectively assists hip flexion and extension during walking. Even with this single-actuator configuration, significant improvements in walking and balance abilities were observed in older adults participating in a walking exercise program, comparable to results achieved with dual-actuator hip exoskeletons^5,14^. The 10MWT walking speed increased from an average of 1.18 ± 0.16 m/s to 1.34 ± 0.14 m/s, reflecting a 14.8% improvement with a mean speed increase of 0.17 m/s. A previous study using a dual actuator reported an increase in speed from 1.21 ± 0.16 m/s to 1.36 ± 0.18 m/s, representing a 12.4% improvement and a 0.15 m/s increase^5^.

For older adults, the minimally clinically important difference (MCID) for the 10MWT is 0.13 m/s, indicating that the exoskeleton intervention produced a statistically and clinically significant improvement in gait speed^14^. Self-selected walking speed in older adults is a crucial indicator, strongly correlated with life expectancy^30^. This finding underscores the potential of exoskeletons for enhancing walking exercise in the elderly. The 6-minute walking speed (6MWT) increased by 10.6% (+ 0.13 m/s), indicating that continued participation in an exercise program also led to improvements in gait endurance. The TUG and FSST completion times were reduced by 24.5% (− 3.13 s) and 19.6% (− 2.96 s), respectively, indicating an improvement in dynamic balance. The arm reach distance measured by the FRT, which indicates the participant’s static balance and flexibility, increased by 5.17 cm. The 5xSTS time, closely associated with lower-body strength, improved by 24.5% (− 4.11 s). Except for the FRT, all other functional assessment items were statistically significant. These results suggest that the proposed robotics hip exoskeleton may be suitable for use as a walking exercise device in older adults.

**Fig. 5:**
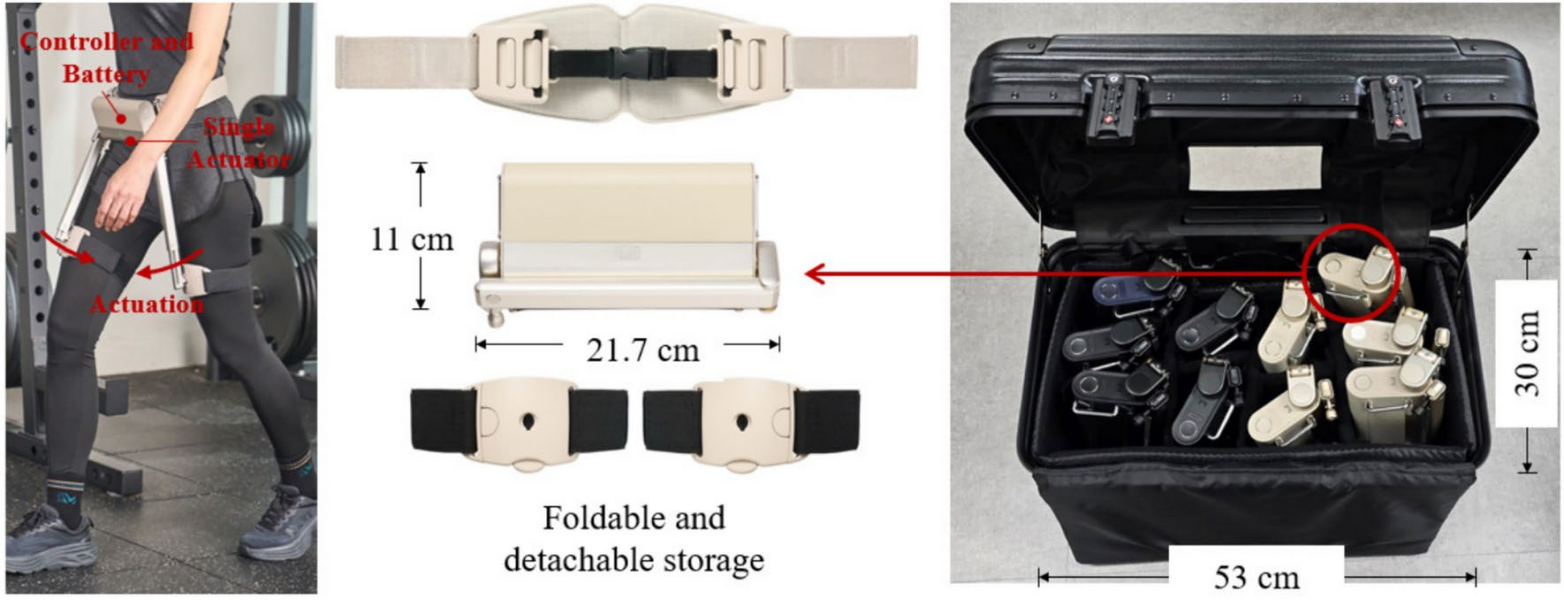
Robotic hip exoskeleton utilizing the WIM demonstrating its portability.

**Fig. 6:**
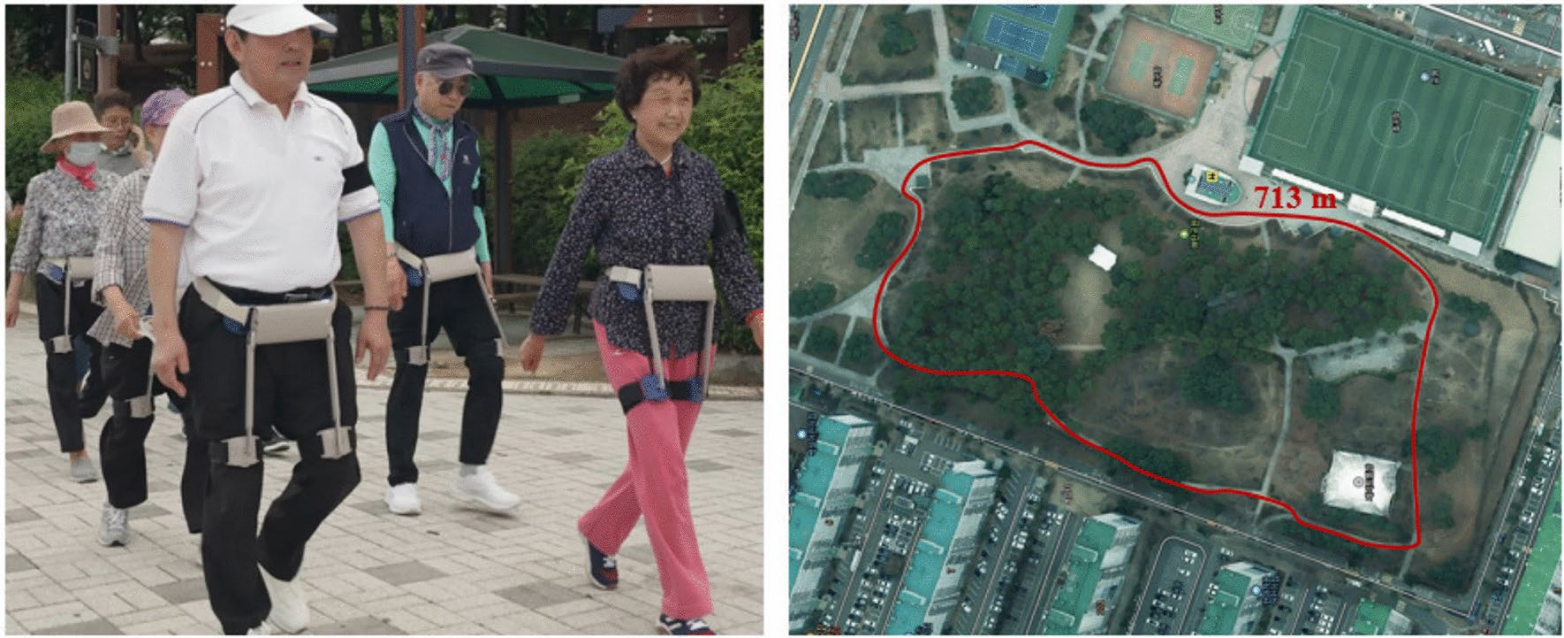
Outdoor group walking exercise using the WIM along park trail routes.

We also observed an improvement in lower-body strength, particularly in ankle strength. The simultaneous assistance of hip flexion and extension is believed to contribute to an increase in stride length, as the proximal hip joint receives direct support. This likely accounts for the observed increase in both stride length and walking speed. Furthermore, increases in stride length and speed may stimulate and engage the muscles involved in ankle dorsiflexion and plantarflexion, resulting in muscle-strengthening effects. Existing research shows that increasing speed through longer stride length, rather than merely by increasing cadence, produces greater lower limb joint moments^31,32^. We propose that redistributing the power ratio across the hip, knee, and ankle, similar to the distribution observed in younger individuals^10^, can enhance load balance, facilitating more energy-efficient and sustained walking. This suggests that hip-assisted walking exercises may improve gait among the elderly and strengthen lower limb muscles. Although not discussed in this study, the device’s capability to apply both resistance and assistance indicates its potential for comprehensive lower-limb strength training (Supplementary Fig. S5).

Additionally, we conducted a supplementary experiment with three young adults to assess the system’s effectiveness in promoting energy-efficient walking. Results demonstrated a reduction in metabolic energy expenditures ranging from 11.6% to 17.3% compared to walking without the device across various ground conditions, including treadmill-level walking, load-carrying walking, and inclined walking. Specifically, during treadmill-level walking, participants exhibited an average metabolic energy expenditure reduction of 13.6%. These findings align with previous research, which reported reductions of approximately 10.6% during flat walking and 15.7% during uphill stair walking assistance for ten industrial workers^33^. This small-scale supplementary study highlights the potential of our single-actuator system. Existing approaches typically result in more complex mechanical systems, despite utilizing a single actuator^16,34^. Notably, there are currently no examples of single-actuator exoskeletons that have successfully achieved reductions in metabolic energy expenditure during walking^1,4^ (Supplementary Table S2).

Outdoor walking exercises likely contributed to the improved effectiveness of the walking exercises. This assumption stems from the observation that, despite shortening the exercise program to four weeks with two sessions per week, similar walking improvements were achieved compared to a previous six-week program with 2-3 sessions per week conducted indoors in hospital corridors and halls^5,14^. This finding supports existing research indicating that outdoor walking exercises offer advantages over indoor walking exercises^35^. In the early stages of the exercise program (weeks 1–2), participants were able to complete only 1–2 laps. However, in the later stages (weeks 3–4), they were able to finish the walking course more quickly, ultimately completing four laps within the designated 30–40 min of walking exercise time.

From a usability perspective, a front-positioned design offers several advantages despite the discomfort associated with weight distribution. In conventional hip exoskeletons, the actuator is often placed next to the hip, and the battery is positioned at the back of the waist^4,14^. This configuration can interfere with natural arm movements during walking and cause discomfort by pressing against the back when sitting. Additionally, control units located near the hip or at the back of the waist may hinder users from easily checking indicators during operation, creating inconvenience, especially for elderly users with reduced flexibility. The proposed design, with buttons and indicators positioned at the front, enables easy operation and comfortable wear for elderly users.

To accommodate various body sizes, including different waist circumferences and leg lengths, the waist-wrapping section utilized a soft material, and an adaptive thigh frame mechanism with adjustable length was employed. Traditional hip exoskeletons typically used a rigid frame that wrapped around the waist and featured fixed-length thigh frames^4,14,36^. In contrast, the proposed hip exoskeleton’s flexible and adjustable design allowed users of varying heights and waist sizes to comfortably wear a single size of the exoskeleton. Notably, the foldable structure significantly enhanced storage and portability, as shown in Fig. 5. In this study, a walking exercise program was conducted outdoors in a park with a group of nine participants, as shown in Fig. 6. The compactness of the device, along with its ease of wear and use, allowed only two trainers to effectively manage the exercise sessions. As shown in Fig. 5, a space that typically stored one or two exoskeletons could now easily accommodate up to ten exoskeleton devices, demonstrating superior storage and transportability.

No control group experiments involving walking exercises without an exoskeleton were conducted, as previous research indicated that walking exercises with an exoskeleton were more effective and provided longer-lasting benefits than those without one^5^. Nevertheless, because the exoskeleton and protocol design used in the walking exercise differ, their conclusions are not directly transferable to this work, even if the control scheme and outcome measures are similar. Likewise, we can only hypothesize that ankle strength gains resulted solely from exoskeleton walking assistance, as the device’s hip power support may have enabled participants to increase and sustain their walking speed. The experiments were coducted on a small number of elderly subjects, who constituted a subset of expected users. These factors can be considered limitations of this study. Future studies will expand testing to a larger sample, diverse age groups, and varied exercise protocols to further validate improvements in gait metabolism, kinematics, dynamics, and muscle strength.

## Methods

The study design and protocol received approval from the Public Institutional Review Board of the National Center for Bioethics Policy (P01-202306-01-038). Interested participants were recruited from a public health center in Suwon, Korea, with prior approval from management and the cooperation of visiting nurses and physical therapists in these institutions. All methods were performed in accordance with relevant guidelines and regulations. Informed written consent was obtained from all participants for participation in this study. Following this, an orientation session was conducted to introduce the use of the hip exoskeleton device and coordinate the participation schedule.

Informed written consent was also obtained from all participants for the publication of identifiable images.

### Anti-phase torque symmetry error

During walking, the torques at the hip, knee, and ankle exhibit periodic over one gait cycle (one stride). We calculated the anti-phase symmetry error using the following equation to compare the anti-phase symmetries of the hip, knee, and ankle torques:

1 $$\begin{aligned} \text{ Anti-phase } \text{ symmetry } \text{ error } = \Sigma _i |\tau (t_i) + \tau (t_i+T/2)| \end{aligned}$$

where $\tau (t_i)$ represents the torque value at the *i*-th sample time and *T* denotes the gait cycle period. For the gait cycle torque trajectory $\tau (t)$ to exhibit perfect anti-phase symmetry, it must satisfy the condition $\tau (t) = -\tau (t+T/2)$. We calculated the anti-phase symmetry error using existing human gait data^37^ on the hip flexion/extension, knee flexion/extension, and ankle dorsiflexion/plantarflexion torque trajectories at four different speeds (Supplementary Fig. S2).

### Participants

To verify the effectiveness of the proposed single actuator-based exoskeleton, the study included nine healthy elderly volunteers (age: 78.6 ± 4.4 years; 7 female/2 male; weight: 56.4 ± 10.1 kg; height: 156.6 ± 7.4 cm) (Supplementary Table S3). Participants were elderly residents from the Suwon area of Korea, recruited based on their ability to walk for more than 30 min. Individuals requiring one-on-one care were excluded from participation due to the group walking exercise conducted on outdoor park trails, as shown in Fig. 6.

### Protocol

*Overview.*  This study employed a single-group, pretest-posttest design to determine the effectiveness of a gait assistance training program using a robotic hip exoskeleton, WIM, in local residential older adults. All participants visited a local park (Maetan Park, Suwon, Korea) twice a week for four weeks, totaling eight visits, to participate in the walking exercise program.

*Group exercise.*  The gait assistance training program lasted for four weeks, with two sessions per week. Participants walked from their homes to the local park according to their exercise schedule, engaging in group exercises simultaneously. A 10-minute warm-up/stretching session without the exoskeleton preceded the main exercise. Following the warm-up, participants wore the exoskeleton for a walking exercise session lasting 30–40 min. A subsequent 10-minute cool-down/stretching session concluded the exercise. The exercise program was supervised by two physical therapists. Exercise intensity was determined by the speed at which participant could walk for 30 min. In later sessions, physical therapists increased the exercise intensity by raising the walking speed. The outdoor park trail used for the program measured 713 m in length and featured flat ground, inclines, and declines, as shown in Fig. 6. The nine devices used for group exercises were presorted and arranged in a small carrier (see Fig. 5), allowing participants to don them at the scheduled exercise time.

*Gait quality and leg muscle strength measure.*  In the 10MWT^38^, subjects were instructed to walk for a distance of 15 m at a comfortable (self-selected) speed. The time taken to cover 10 m was measured, excluding the initial 2.5 m for acceleration and the final 2.5 m for deceleration. The recorded time in seconds was then converted to speed (m/s). The 6MWT^39^ measures the distance an individual can walk at a normal pace within 6 min. This test evaluates aerobic capacity and endurance, providing insights into overall functional mobility and exercise tolerance. A greater distance covered indicates better aerobic fitness. The Short Physical Performance Battery (SPPB)^40^ is a tool developed by the National Institute on Aging in the United States to assess lower extremity function. It includes three components: an upright balance test, a walking speed test, and a chair rise test. Each component was scored on a scale from 0 to 4. The upright balance test includes side-by-side, semi-tandem, and tandem stances, each maintained for a minimum of 10 s. The walking speed test measures the time taken to walk 4 m at a normal pace, while the chair-rise test measures the time required to stand up from and sit down into a chair. A higher total SPPB score indicated better physical performance. The TUG test^41^ assessed dynamic balance and mobility by measuring the time taken to rise from a chair, walk a 6 m round trip, and return to a seated position. Shorter completion times indicated better balance and mobility. The FSST^42^ measured dynamic balance and mobility using a cross-shaped layout created with four 90 cm rods on the floor. Participants began in square 1 and moved clockwise through squares 2, 3, 4, and back to square 1, then counterclockwise through squares 4, 3, 2, and back to square 1. Again, shorter times indicated better balance and mobility. The Five Times Sit-to-Stand Test (5xSTS)^43^ evaluated lower body strength and mobility by timing how quickly a participant could stand up and sit down five times. Shorter times reflected better strength and mobility. The Functional Reach Test (FRT)^44^ assessed balance by measuring the maximum distance participants could reach with their arms fully extended in front of them. A longer distance (cm) indicated improved dynamic balance ability.

Muscle strength was measured at the hip, knee, and ankle using a digital dynamometer, Micro FET2 (Hoggan Scientific, LLC, USA) (range: 0-136 kgf). Assessments were conducted in both seated and supine positions on a bed, adhering to a standardized protocol (Supplementary Fig. S6) that included six strength-related items: hip flexion, hip extension, knee flexion, knee extension, dorsiflexion, and plantar flexion. The strengths of the left and right legs were measured separately and averaged. Higher values indicate greater muscle strength. To assess changes in gait quality and lower body strength before and after training, gait/balance ability tests were conducted without the exoskeleton. An experienced physical therapist performed all assessments.

### Robotic hip exoskeleton

The robotic hip exoskeleton, termed WIM used a single electric motor and angle sensor for generating walking assistance torque. Supplementary Table S4 provides the dimensions and hardware specifications of the WIM. When the thigh frame is folded, the main body volume measures 21.7 $\times$ 11.0 $\times$ 5 cm, housing the electric motor, battery, and controller. The battery capacity is 3.35 Ah, enabling continuous use for approximately 2 h with a peak assist torque of 4–5 Nm. The main body is designed in a single size, while the waist belt and thigh attachments are available in small and medium sizes, accommodating waist circumferences ranging from 26 inches to 36 inches (approximately 66.04–91.44 cm). The stroke length of the adaptive frame varies from a minimum of 160 mm to a maximum extended length of 350 mm, allowing compatibility with diverse heights and leg lengths. The WIM hip exoskeleton features 11 degrees of freedom, comprising 3 degrees of freedom at the hip, 2 degrees of freedom in the adaptive frame, and 6 degrees of freedom in the thigh connector^33^ (Supplementary Fig. S8). Among these, only one degree of freedom is attributed to the active joint of the hip, while the thigh frames in the sagittal plane are capable of movement in both widening and narrowing directions. For a detailed explanation of the single actuation mechanism, refer to the Supplementary Method and Figs. S8–S10. The use of low-reduction-ratio gears with minimal mechanical inertia and friction enables backdrivability, allowing for natural walking movements even when the device is turned off. The hip angle difference is sensed by counting rotations via Hall sensors integrated into the motor control, and the torque value is estimated based on the sensed current. The WIM control algorithm and hardware were implemented in C++ on the STM32F405RG microcontroller.

The generation of assistance torque in the robotic hip exoskeleton was based on the difference in hip angles sensed during walking. Supplementary Figs. S3 and S7 illustrate the proposed Adaptive DOFC controller used for gait assistance. A state variable, $s_{0}$, was defined to represent the gait behavior, utilizing the current hip angle. The original state value was then smoothed through an exponential moving average filter, incorporating the current raw state data $s_{0}$ and the previously smoothed state data $s_{0,prv}$ ($s_0 \rightarrow \alpha s_0 + (1-\alpha ) s_{0,prv}$). Hip state data from the most recent second were stored in a state-trajectory buffer, and real-time torque was generated based on the discrete data retained in this buffer. The decision to use the state value stored at a particular position was made adaptively, depending on the degree of fluctuation in the state trajectory recorded in the buffer. The fluctuation level of the state trajectory over the past second was determined by the length of the discrete state values. Specifically, if the walking speed increased, the value lengthened; conversely, if the individual remained stationary, the state trajectory length over 1 s became zero (as detailed in the Supplementary Method). Finally, assistive or resistive torque was produced selectively through positive or negative gains. The user had the ability to adjust the asymmetry factor, which enabled the generation of asymmetric torque and gain to control intensity. In this study, an asymmetry value of zero was employed, and gain values ranging from 6 to 13 (with a maximum assistive torque of 8.5 Nm) were applied, as shown in Fig. 4b.

### Statistics

All statistical analyses were performed using MATLAB (MathWorks, Natick, MA, USA). Results are reported as the mean ± standard deviation of the mean ± SD. Functional performance and muscle strength were assessed both before and after the gait assistance training program. The same researcher conducted all pre- and post-assessments to ensure consistency. Mean differences in functional performance and muscle strength before and after the program were compared using paired t-tests. Two-sided *p*-values less than 0.05 were considered statistically significant. Pre- and post-program data were assessed for normality using the Shapiro-Wilk test. All variables, except for the ankle plantarflexor in the MMT data, met the normality assumption. For non-normally distributed data, the Wilcoxon signed-rank test was employed.

### Supplementary test, n = 3

In a separate supplementary test for metabolic measurements, three young adult subjects participated to determine the reduction in metabolic energy under various ground conditions (age: 42.0 ± 1.7 years; weight: 65.7 ± 2.5 kg; height: 168.3 ± 7.6 cm, mean ± SD). Three participants participated in the treadmill walking experiment with the treadmill speed set to 4 km/h. Detailed information on all participants is available in the attachment (Supplementary Table S7). Only the third participant participated in the load carrying and incline walking experiments. During the load-carrying experiment, the backpacks they wore weighed 20 kg, and the treadmill speed was set at 4 km/h. In the inclined walking experiment, the treadmill speed was set at 3 km/h with an incline of 12% (7 degrees). We compared the difference in metabolic energy expenditure during assisted walking with an exoskeleton device to normal walking without it. The metabolic energy measurements were performed as follows: First, the participants stood for 5 min (and again at the end of the experiment) to obtain the average baseline, and the walking data were subtracted to determine the net metabolic rate. They then walked without the exoskeleton (no exoskeleton) for 6 min (and again immediately before the final 5-min stand) to obtain the average metabolic rate under normal walking conditions. Subsequently, the subjects wore the exoskeleton and walked with assistance on a treadmill. The average of the last 3 min of each condition was calculated to represent the metabolic rate expended under these conditions. A K5 breath-by-breath portable metabolic system (COSMED, Rome, Italy) was used to measure metabolic energy expenditure.

## Supplementary Information

Supplementary Information.

## Data Availability

All data generated and analyzed in this study are included in this published article and its Supplementary Information.
