# Supplementary material for: Ultra-lightweight robotic hip exoskeleton with anti-phase torque symmetry for enhanced walking efficiency
Source: Sci Rep. 2025 Mar 29;15:10850. doi: 10.1038/s41598-025-95599-2 (PMC11954954; doi:10.1038/s41598-025-95599-2)
Supplement: Supplementary file 1 — Supplementary Information. [file 41598_2025_95599_MOESM1_ESM.pdf]

Supplementary Information for

Ultra-Lightweight Robotic Hip Exoskeleton with Anti-Phase Torque Symmetry for Enhanced Walking Efficiency

Bokman Lim[1*], Byungjune Choi[1], Changhyun Roh[1], Jewoo Lee[1], Yong-Jae Kim[1,2], and Younbaek Lee[1]

[1]Robot R&D Team, WIRobotics, Yongin 16942, Korea
[2]School of Electrical, Electronics & Communication Engineering, Korea University of Technology and Education, Cheonan 31253, Korea
[*]bokman.lim@wirobotics.com

This PDF file includes:

Assistance Method

Figures S1 to S10

Tables S1 to S7

1 Assistance Method

The Adaptive Delayed Output Feedback Control (Adaptive DOFC) method is used for gait assistance. Figure 1 and Supplementary Fig. S3 show the assistance algorithm applied to the We Innovate Mobility (WIM) system. This adaptive output feedback control method does not include a gait phase/event estimator or references for generating assistive torque. The assistive torques were generated and applied immediately following the movement of the user by updating the change in hip state at every control period (100 Hz). The higher-level desired torque update (regeneration) operates at 100 Hz, while the lower-level current-based torque control runs faster at 1000 Hz. To allocate computing resources for separate high-level algorithms (e.g., gait performance estimation), we configured the torque generation algorithm proposed in this study to run at 100 Hz, which is slower than the low-level control cycle. Supplementary Fig. S3 shows the control flow of the Adaptive DOFC-based assistance.

The hip state trajectory data $s = \{s_0, s_1, \cdots, s_N\}$ from the recent one-second interval was stored in a state trajectory buffer. The current hip state is determined by the asymmetry factor a and the current hip difference angle q_0.

$$s_0 = -sin(q_0/2) - sin(q_0/2 - a) \tag{1}$$

To generate left-right symmetric assist torque, you can set $s_0 = -2sin(q_0/2)$. Torque is generated in real time based on the discrete data stored in the buffer. The control state s_0 smoothed (filtered) by passing through a first-order low-pass filter.

$$s_0 \rightarrow \alpha s_0 + (1-\alpha)s_{0,prv} \tag{2}$$

The assistive and resistive torques were selectively generated using the weighted summation of the selected states.

$$\tau_0 = \kappa \Sigma w_i s_i \tag{3}$$

where a positive gain ($\kappa > 0$) denotes assistive torque, a negative gain ($\kappa < 0$) denotes resistive torque. When using the two selected states, s_i and s_{i+1}, applied in this study, τ takes the form $\tau_0 = \kappa(w_i s_i + w_{i+1}s_{i+1}), w_i = 0.8, w_{i+1} = 0.2$.

User-adjustable parameters include the asymmetry factor, which can produce asymmetric torque, and the gain to control intensity. In this study, an asymmetry value of 0 was used, and a gain of 6–13 (with a maximum torque of 4.5–8.5 Nm) was applied. The selected hip state position and the filter parameter α are adaptively adjusted by calculating the state trajectory distance in real time. The state trajectory displacement d_0 is calculated by summing the squared differences between the hip state values stored in the state trajectory memory buffer, expressed as: $d_0 = \Sigma_{i=0}^{N-1}\sqrt{(s_i - s_{i+1})^2}$. Since the motion state values are generated and stored at regular time intervals, a larger state trajectory displacement indicates that the user has made a rapid change in motion. In other words, as the state-trajectory displacement d_0 increases, the selection position of the motion state value moves closer to the first storage position (0) in the memory array of the state-trajectory memory buffer. Conversely, a smaller state trajectory displacement causes the motion state value selection position to move closer to the last storage position (N) in the memory array. This relationship implies that the more rapidly the user's motion changes, the more recent the motion state values used to determine the assistive force. The filter parameter α, similar to the hip state selection, is adaptively determined in proportion to the state trajectory displacement, with values ranging from 0.05 to 0.10. In this study, the initial α value used was 0.05.

The adaptive delayed output feedback control (Adaptive DOFC) algorithm can be seen as a generalized version of existing DOFC[1,2,3] methods. By minimizing the reduction in responsiveness caused by delays in gait phase estimation time/accuracy, it maintains the generality of the original DOFC while allowing fast and stable responses not only during normal-speed walking but also during high-speed walking. In the case of the Adaptive DOFC, even as walking speed increases, the timing of the peak torque shifts adaptively, allowing for the generation and transfer of appropriate positive power during high-speed walking, as shown in Supplementary Fig. S7. In addition, this approach can be applied to exoskeletons that utilize a single actuator and sensor. Despite employing a single actuator, it offers versatile functionality capable of applying various interaction torques. As shown in Supplementary Fig. S5, assistance and resistance torques for flexion and extension can be effectively applied. Supplementary

Fig. S4 shows that an asymmetric assistance torque for the left or right side can be generated using a single actuator. While walking at 4 km/h, the asymmetry factor increased by 0.05 per cycle, ranging from -0.5 to 0.5. When the asymmetry factor a is negative, the right step (right flexion, left extension) receives stronger assistance; conversely, when the asymmetry factor a is positive, the left step (left flexion, right extension) is assisted more strongly. Similarly, asymmetric resistance torque for the left and right sides can be generated using a negative gain. This demonstrates the versatility of the control algorithm in generating a wide range of interaction torques with a single actuator.

1.1 Single-Motor Actuation Mechanism

Supplementary Fig. S8 illustrates the overall mechanical structure of the WIM device. The actuator is fixed inside the cylindrical actuator frame, which can rotate freely, causing the actuator to rotate with it (Supplementary Fig. S8). It consists of a cylindrical rotary motor that changes rotation direction. The motor shaft connects to the left adaptive sliding frame on the user's left thigh, while the opposite end of the actuator frame, secured to the motor housing, connects to the right adaptive sliding frame on the right thigh (Supplementary Fig. S9). Due to this single-actuator mechanical structure, the output generated by the actuator is transmitted as assistive force to the left thigh through the connecting member, while a reaction force against the actuator's rotational force acts on the right thigh, generating assistive force there as well (Supplementary Fig. S9).

The single actuator functions based on the angle difference between the legs and incorporates a differential structure that allows free rotation within the main housing (Supplementary Fig. S10). This design prevents users from experiencing unwanted rotational forces, ensuring comfort even when the main body's posture changes during level walking, stair climbing, or sitting (Supplementary Fig. S10). For this single differential actuation, a Maxon EC-i30 brushless DC motor was used with a Maxon GPX planetary gear as a reducer. The maximum driving torque is approximately 8.5 Nm, and assistive torque is generated only during walking, allowing free movement in other motions through differential actuation.

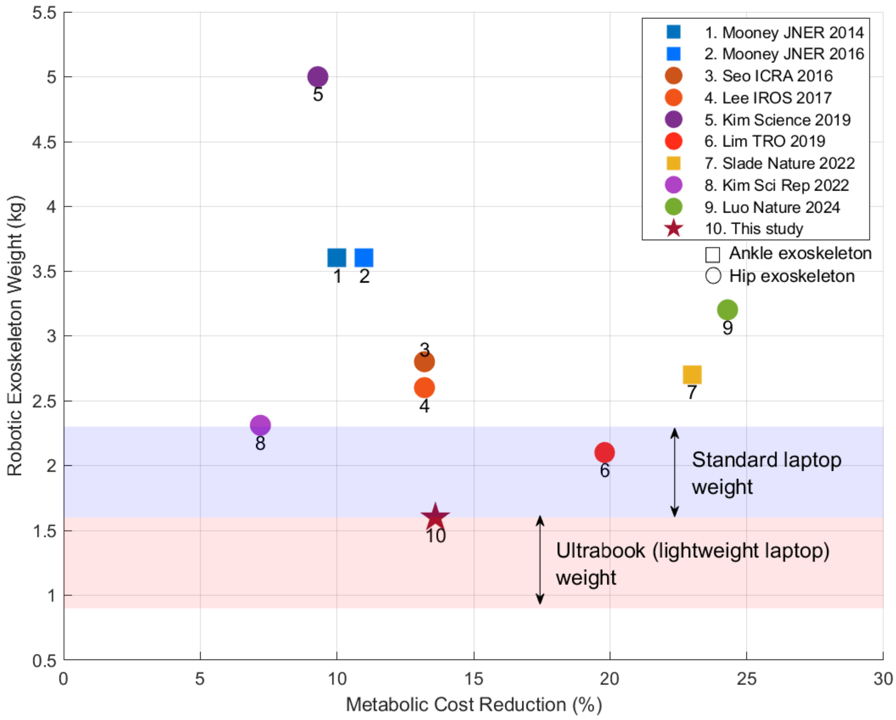

Figure S1. Comparison of exoskeleton weight and reduction rate of walking metabolic energy.

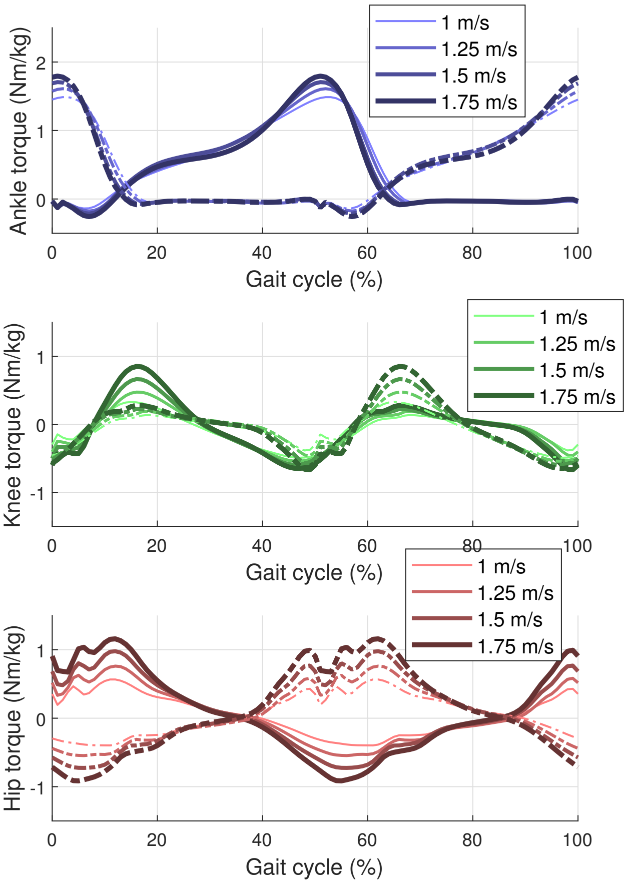

Figure S2. Human hip, knee, and ankle torque trajectories for various walking speeds. Adapted and redrawn from the human gait data[4] for anti-phase symmetry comparison.

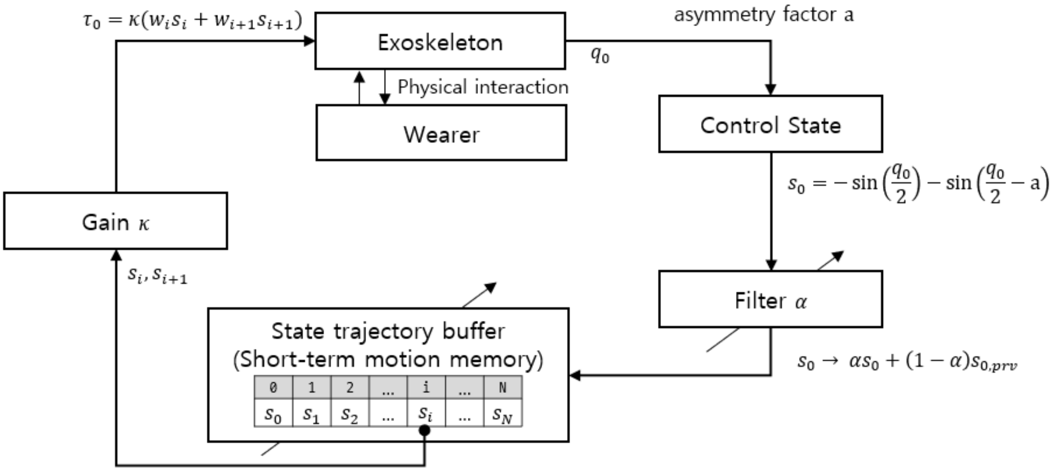

Figure S3. Adaptive delayed output feedback controller.

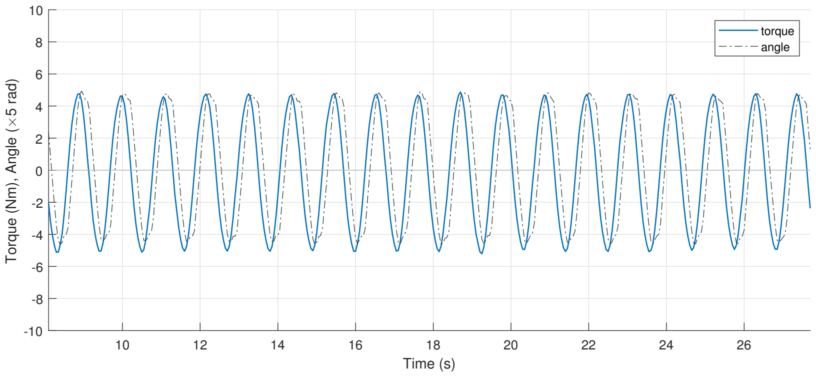

(a) Symmetric assistance

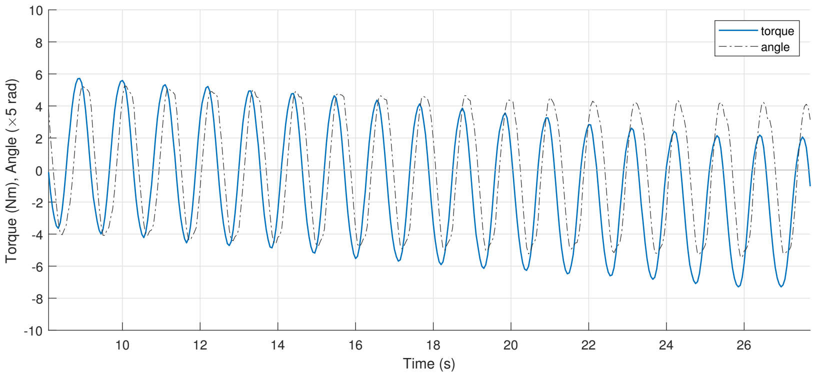

(b) Asymmetric assistance

Figure S4. Comparison of symmetric and asymmetric assistance.

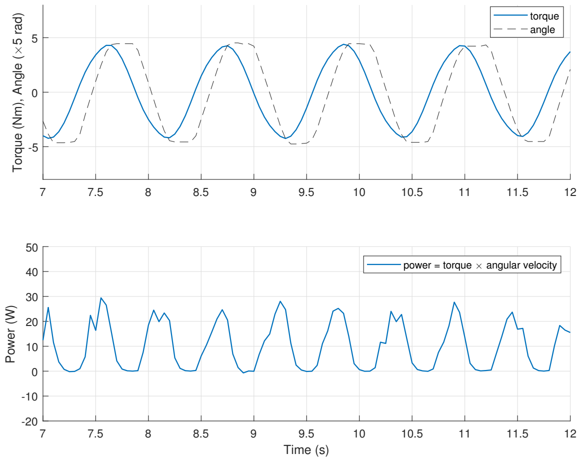

(a) Assistance

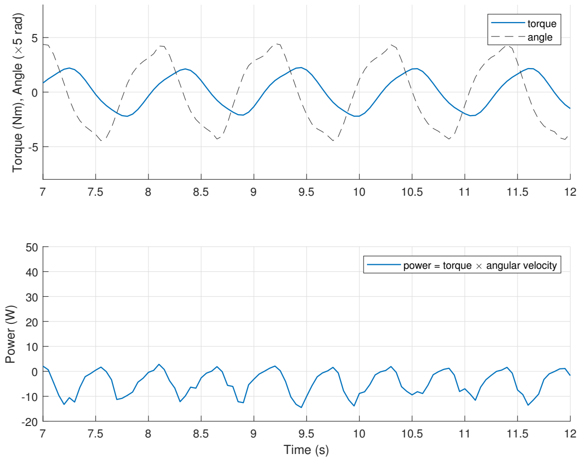

(b) Resistance

Figure S5. Comparison of assistance and resistance during 4 km/h treadmill walking. In steady-speed treadmill walking, gait angles and real-time torque generation remain consistent.

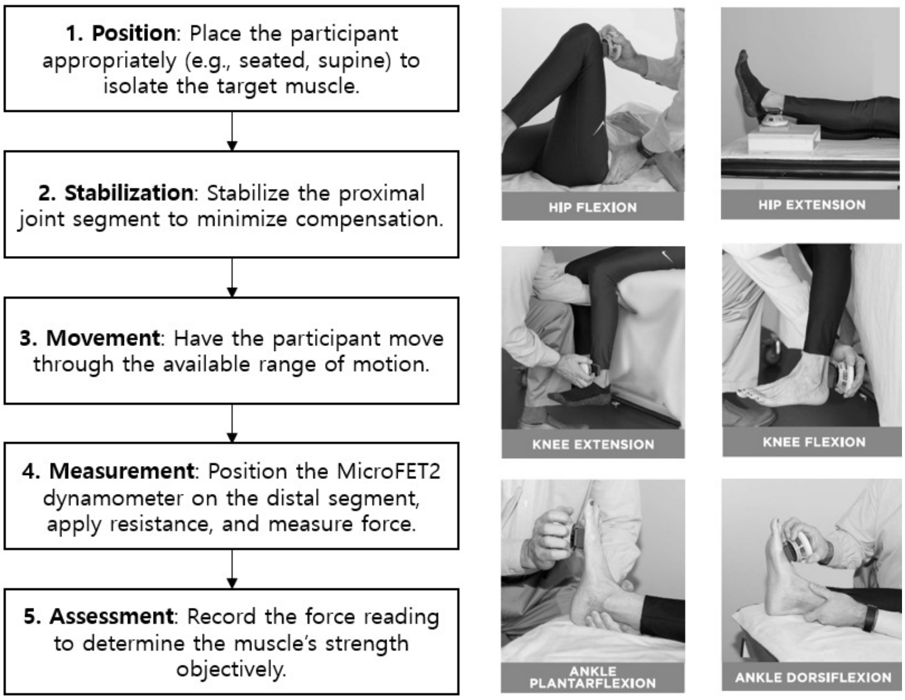

Figure S6. Overview of the MicroFET2 Muscle Strength Testing Protocol.

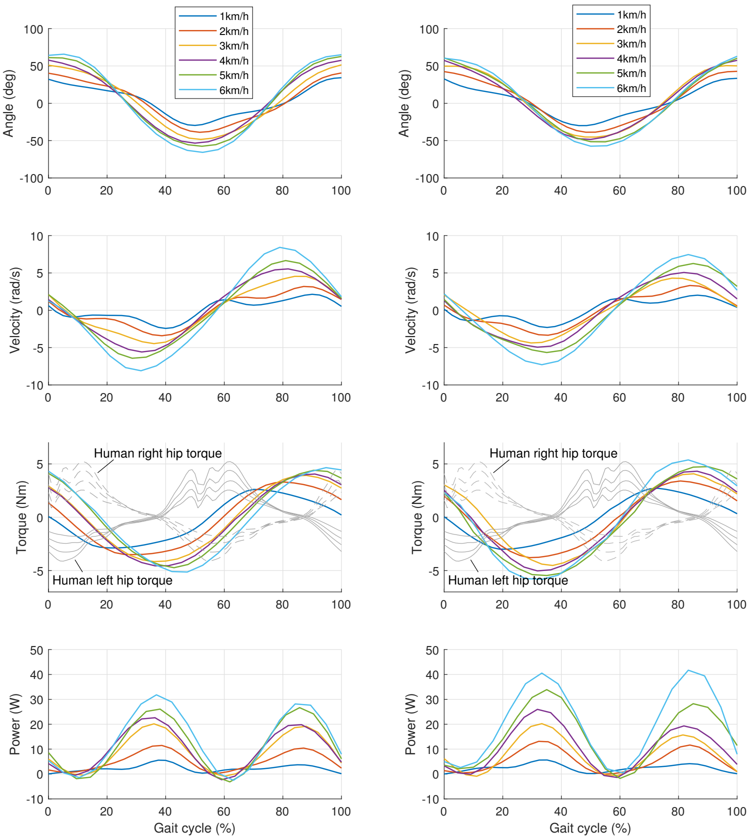

(a) DOFC (b) Adaptive DOFC

Figure S7. Comparison of device angle, angular velocity, torque, and power trajectories at different speeds. Gait cycle 0% corresponds to right heel contact and 100% to the subsequent right heel contact. Notice that the device angle, angular velocity, torque, and power refer to values measured at the device posture for each gait phase. For example, at 0% of the gait cycle, the left hip is maximally extended behind the body while the right hip is maximally forward. Left hip extension is defined as the positive rotation direction and flexion as the negative direction. The gray solid and dotted lines denote the human biological hip torque trajectory at 3.5, 4.5, 5.5, and 6.5 km/h walking (scaled from [4] for comparison).

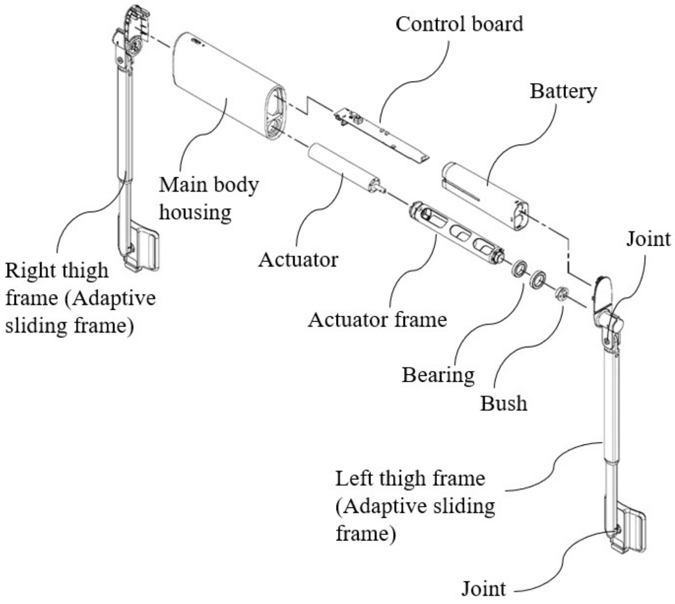

Figure S8. Exploded view of the WIM divice.

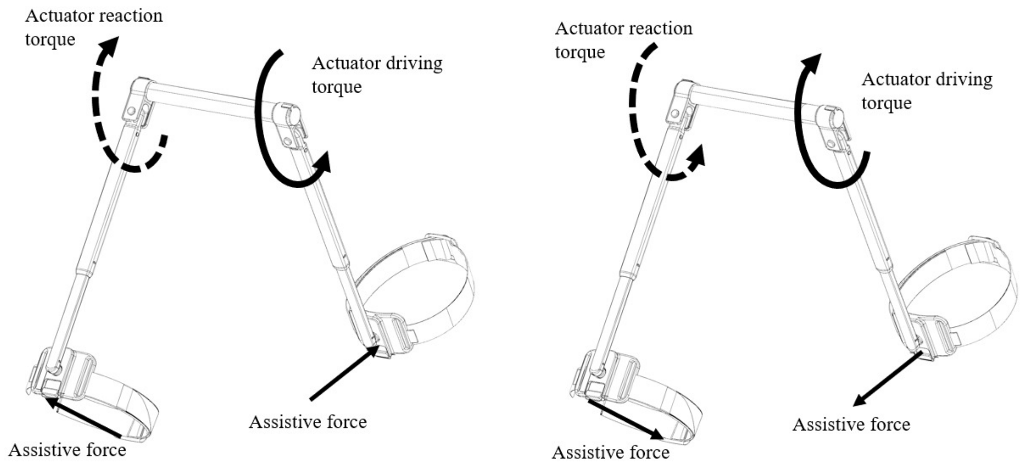

(a) Left extension and right flexion assistance

(b) Left flexion and right extension assistance

Figure S9. Single motor-based double hip actuation mechanism.

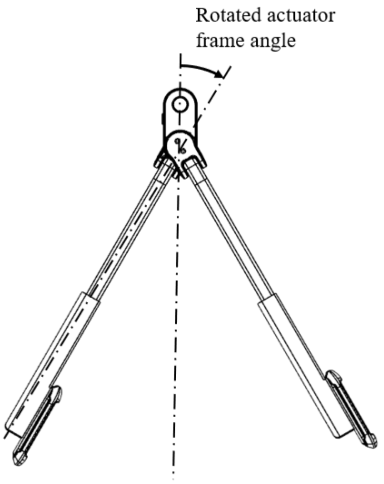

(a) Level-ground walking

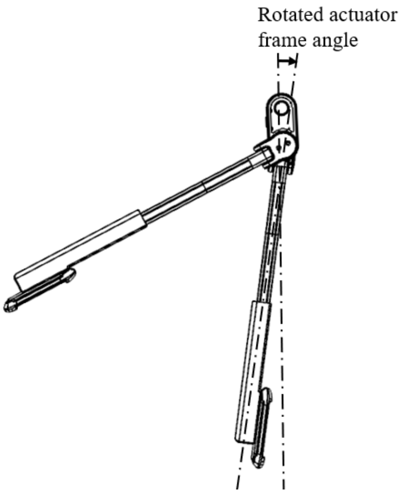

(b) Stair ascent walking

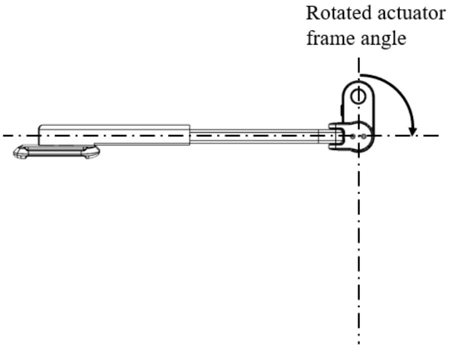

(c) Seated position

Figure S10. Rotated actuator frame angle in various situations.

Subject (walking condition)	No Exo (kcal/min)	Exo (kcal/min)	rNMR (%)
S1 (level at 4 km/h)	2.75	2.43	11.8
S2 (level at 4 km/h)	3.10	2.56	17.3
S3 (level at 4 km/h)	2.80	2.48	11.6
Mean±SD	2.88±0.19	2.48±0.05	13.57±3.23
S3 (loadcarry 20 kg at 4 km/h)	4.60	4.10	10.8
S3 (ramp 16% grade at 3 km/h)	5.00	4.25	14.8

Table S1. Metabolic measurement results for gait assistance under various conditions with three young adults. No Exo: net metabolic rate during walking without exo; Exo: net metabolic rate during walking with exo; rNMR: reduced net metabolic rate by assistance.

No.	Mass (kg)	rNMR (%)	Joint	Actuator(s)	Assistance	Reference
1	3.6	10	Ankle	Dual	Plantarflexion	Mooeny14[5]
2	3.6	11	Ankle	Dual	Plantarflexion	Mooeny14[6]
3	2.8	13.2	Hip	Dual	Flexion & Extension	Seo16[7]
4	2.6	13.2	Hip	Dual	Flexion & Extension	Lee17[8]
5	5.0	9.3	Hip	Dual	Extension	Kim19[9]
6	2.1	19.8	Hip	Dual	Flexion & Extension	Lim19[1]
7	4.3	None	Hip	Single	Flexion	Hsieh20[10]
8	2.2	None	Hip	Single	Flexion	Tricomi21[11]
9	2.7	23	Ankle	Dual	Plantarflexion	Slade22[12]
10	2.31	7.2	Hip	Dual	Flexion	Kim22[13]
11	3.2	24.3	Hip	Dual	Flexion & Extension	Luo24[14]
12	1.6	13.6	Hip	Single	Flexion & Extension	This work

Table S2. Comparison of exoskeleton total weight and reduction in walking metabolic energy. rNMR: reduced net metabolic rate by assistance.

No.	Age (yrs)	Sex	Height (cm)	Weight (kg)
1	87	Female	156.0	55
2	78	Female	158.0	57
3	78	Female	156.0	52
4	76	Female	158.0	47
5	79	Female	145.0	55
6	82	Female	150.0	45
7	71	Male	171.0	73
8	76	Female	153.0	51
9	80	Male	162.0	73
Mean±SD	78.6±4.4	-	156.6±7.4	56.4±10.1

Table S3. Information on elderly participants.

Overall size	21.7 cm × 11.0 cm × 5.5 cm
Total weight	1.6 kg (including battery and fasteners)
Operating time per charge	Approximately 2 h
Battery	Lithium-ion battery, 14.4 Vd.c., 3.35 Ah
Applicable body size	Main body - one size
	Waist or thigh fastener - two sizes (waist 26'–36')
Adaptive thigh frame stroke	160 – 350 mm

Table S4. Hip exoskeleton WIM size dimensions and hardware specifications.

Subject	10MWT (s)		6MWT (m)		TUG (s)		FSST (s)		5XSTS (s)		FRT (cm)		SPPB	
Number	Pre	Post	Pre	Post	Pre	Post	Pre	Post	Pre	Post	Pre	Post	Pre	Post
1	1.19	1.29	1.27	1.38	13.66	9.67	18.52	13.43	19.86	11.83	20	28	8	11
2	1.33	1.56	1.31	1.40	11.40	7.84	13.91	10.41	13.22	5.70	24	22	11	12
3	1.01	1.23	1.14	1.31	14.77	8.53	21.92	16.11	23.70	9.52	10	30	9	12
4	1.20	1.39	1.32	1.52	10.97	7.98	11.48	9.50	13.31	9.87	25	27	11	12
5	1.20	1.26	1.19	1.30	11.46	8.75	15.35	12.15	14.00	14.36	19	26	10	10
6	0.85	1.13	1.00	1.26	16.79	10.97	14.37	12.07	18.25	20.73	19	21	6	9
7	1.27	1.52	1.31	1.38	8.67	7.30	9.83	8.73	14.10	10.41	36.5	40	10	12
8	1.23	1.33	1.28	1.36	9.14	7.73	10.42	8.66	9.68	8.49	30	28	12	12
9	1.32	1.39	1.32	1.37	8.72	8.62	13.05	11.12	11.72	9.92	22	30	11	12
Mean	1.18	1.34	1.24	1.36	11.73	8.60	14.32	11.35	15.32	11.20	22.8	28.0	9.78	11.33

Table S5. Changes in functional outcomes before (Pre) and after gait exercise sessions (Post) for all nine older adults.

Subject	Hip Flx (kgf)		Hip Ext (kgf)		Knee Flx (kgf)		Knee Ext (kgf)		Ankle DF (kgf)		Ankle PF (kgf)	
Number	Pre	Post	Pre	Post	Pre	Post	Pre	Post	Pre	Post	Pre	Post
1	7.9	8.3	8.5	9.6	11.1	11.9	15.7	12.1	9.0	12.0	8.7	12.8
2	10.3	12.1	11.4	9.8	14.4	14.8	14.5	18.1	7.6	11.5	11.3	15.2
3	9.5	9.1	10.4	8.3	9.5	9.0	12.8	11.7	10.0	11.8	15.6	13.7
4	10.7	8.0	8.7	10.0	11.0	10.7	14.7	14.5	9.4	13.5	12.7	18.7
5	8.2	8.6	9.7	8.9	9.9	12.7	9.4	12.8	7.1	17.4	9.1	13.1
6	6.2	7.3	6.6	7.7	5.8	6.9	6.2	5.8	3.5	8.7	5.6	10.6
7	13.8	16.6	11.9	15.3	13.9	13.4	10.5	14.8	8.7	19.4	10.9	17.3
8	11.2	10.6	11.5	11.4	12.4	12.8	11.1	12.7	8.8	16.4	11.5	15.2
9	8.7	15.8	17.6	11.8	14.1	13.3	11.2	12.9	10.5	13.5	11.3	17.0
Mean	9.58	10.68	10.66	10.28	11.33	11.69	11.77	12.81	8.27	13.78	10.71	14.81

Table S6. Results of muscle strength measurements before (Pre) and after gait exercise sessions (Post) for all nine older adults. Flx: flexion; Ext: extension; DF: dorsiflexion; PF: plantarflexion

Subject	Age (yrs)	Sex	Height (cm)	Weight (kg)
1	41	Male	175	66
2	41	Male	170	63
3	44	Male	160	68
Mean±SD	42.0±1.7	-	168.3±7.6	65.7±2.5

Table S7. Young adult participants' information for the supplementary test.

Supplementary References

1. Lim, B. et al. Delayed output feedback control for gait assistance with a robotic hip exoskeleton. IEEE Transactions on Robotics 35, 1055–1062 (2019).

2. Lim, B. et al. Delayed output feedback control for gait assistance and resistance using a robotic exoskeleton. IEEE Robotics Autom. Lett. 4, 3521–3528 (2019).

3. Lim, B. et al. Parametric delayed output feedback control for versatile human-exoskeleton interactions during walking and running. IEEE Robotics Autom. Lett. 8, 4497–4504 (2023).

4. Wang, J. M., Hamner, S. R., Delp, S. L. & Koltun, V. Optimizing locomotion controllers using biologically-based actuators and objectives. ACM Transactions on Graph. (TOG) 31, 1–11 (2012).

5. Mooney, L. M., Rouse, E. J. & Herr, H. M. Autonomous exoskeleton reduces metabolic cost of human walking. J. NeuroEngineering Rehabil. 11, 1–6 (2014).

6. Mooney, L. M. & Herr, H. M. Biomechanical walking mechanisms underlying the metabolic reduction caused by an autonomous exoskeleton. J. neuroengineering rehabilitation 13, 1–12 (2016).

7. Seo, K., Lee, J., Lee, Y., Ha, T. & Shim, Y. Fully autonomous hip exoskeleton saves metabolic cost of walking. In 2016 IEEE International Conference on Robotics and Automation (ICRA), 4628–4635 (IEEE, 2016).

8. Lee, Y. et al. A flexible exoskeleton for hip assistance. In 2017 IEEE/RSJ International Conference on Intelligent Robots and Systems (IROS), 1058–1063 (IEEE, 2017).

9. Kim, J. et al. Reducing the metabolic rate of walking and running with a versatile, portable exosuit. Science 365, 668–672 (2019).

10. Hsieh, M.-H. et al. Single-actuator-based lower-limb soft exoskeleton for preswing gait assistance. Appl. Bionics Biomech. 2020, 5927657 (2020).

11. Tricomi, E. et al. Underactuated soft hip exosuit based on adaptive oscillators to assist human locomotion. IEEE Robotics Autom. Lett. 7, 936–943 (2021).

12. Slade, P., Kochenderfer, M. J., Delp, S. L. & Collins, S. H. Personalizing exoskeleton assistance while walking in the real world. Nature 610, 277–282 (2022).

13. Kim, J. et al. Reducing the energy cost of walking with low assistance levels through optimized hip flexion assistance from a soft exosuit. Sci. reports 12, 11004 (2022).

14. Luo, S. et al. Experiment-free exoskeleton assistance via learning in simulation. Nature 630, 353–359 (2024).
